# Survival after repeated surgery for lung cancer with idiopathic pulmonary fibrosis: a retrospective study

**DOI:** 10.1186/s12890-018-0703-8

**Published:** 2018-08-10

**Authors:** Seijiro Sato, Yuki Shimizu, Tatsuya Goto, Akihiko Kitahara, Terumoto Koike, Hiroyuki Ishikawa, Takehiro Watanabe, Masanori Tsuchida

**Affiliations:** 1 Division of Thoracic and Cardiovascular Surgery, Niigata University Graduate School of Medical and Dental Sciences, 1-757 Asahimachi-dori, Chuo-ku, Niigata-shi, Niigata, 951-8510 Japan; 2 Department of Radiology and Radiation Oncology, Niigata University Graduate School of Medical and Dental Sciences, Niigata, Japan; 3 Department of Thoracic Surgery, National Hospital Organization Nishi-Niigata Chuo National Hospital, Niigata, Japan

**Keywords:** Lung cancer, Idiopathic pulmonary fibrosis, Repeated surgery, Acute exacerbation, Percent vital capacity

## Abstract

**Background:**

Patients with idiopathic pulmonary fibrosis (IPF) have a high risk of developing lung cancer, but few studies have investigated the long-term outcomes of repeated surgery in such patients. The purpose of this study was to evaluate the surgical outcomes of repeated lung cancer surgery in patients with IPF.

**Methods:**

From January 2001 to December 2015, 108 lung cancer patients with IPF underwent pulmonary resection at two institutions; 13 of these patients underwent repeated surgery for lung cancer, and their data were reviewed.

**Results:**

The initial procedures of the 13 patients were lobectomy in 8, segmentectomy in 2, and wedge resection in 3. The subsequent procedures were wedge resection in 10 and segmentectomy in 3. The clinical stage of the second tumor was stage IA in 12 and stage IB in 1. Postoperatively, 3 patients (23.1%) developed acute exacerbation (AE) of IPF and died. The rate of decrease in percent vital capacity was significantly higher in patients with AE than in those without AE (*p* = 0.011). The 3-year overall survival rate was 34.6%. The causes of death were cancer-related in 7, AE of IPF in 3, and metachronous lung cancer in 1.

**Conclusions:**

Despite limited resection, a high incidence of AE was identified. The early and long-term outcomes of repeated surgery in lung cancer patients with IPF were poor because of the high risk of AE of IPF and lung cancer recurrence. Long-term intensive surveillance will be required to determine whether surgical intervention is justified in patients with multiple primary lung cancers and IPF.

**Electronic supplementary material:**

The online version of this article (10.1186/s12890-018-0703-8) contains supplementary material, which is available to authorized users.

## Background

The incidence of lung cancer is higher in patients with idiopathic pulmonary fibrosis (IPF) than in the general population; the relative risk of lung cancer in such patients ranges from 6 to 17% [1, 2]. In the general population, the likelihood of a new primary lung cancer developing after complete resection for an initial lung cancer has been reported to be 1% to 2% per patient per year [3, 4]. On the other hand, in patients with IPF, the cumulative rate of developing lung cancer has been reported to increase as the duration of follow-up increased (3.3%, 15.4%, and 54.7% at 1, 5, and 10 years, respectively) [5].

Thoracic surgeons, as well as medical and radiation oncologists, must often make difficult decisions in treating lung cancer patients with IPF because of the poor prognosis of IPF itself and the complications, such as acute exacerbation (AE), which arise after each intervention [5–9]. Several previous studies demonstrated a median survival time of 2 to 3 years after diagnosis in patients with IPF [10–14]. Sato and colleagues [15] reported that 9.3% of lung cancer patients with IPF developed AE after pulmonary resection. To estimate the risk of surgery, they proposed a risk score using clinical characteristics and surgical procedures. However, repeated surgical intervention was not included as a risk factor. With regard to the outcome of surgical intervention, several studies [9, 16, 17] reported 5-year survival rates of about 40% to 60% for stage I patients; therefore, surgical treatment for lung cancer with concomitant IPF might not be an absolute contraindication, as long as the patients are carefully selected. For lung cancer patients with IPF who undergo surgical interventions, many challenging problems, such as AE of IPF and high rates of second and third primary cancers, have been cited; however, to the best of our knowledge, there have been no studies focusing on the incidence of postoperative AE of IPF and the long-term outcome after a second pulmonary resection***.*** Thus, the purpose of this study was to evaluate the outcomes and risks after a second pulmonary resection and to elucidate the implications of surgical interventions in lung cancer patients with IPF.

## Methods

The medical records of all lung cancer patients admitted from 2001 to 2015 to the Division of Thoracic and Cardiovascular Surgery at Niigata University Hospital and the Department of Thoracic Surgery at Nishi-Niigata Chuo National Hospital were retrospectively reviewed; patients diagnosed with IPF before surgical treatment for lung cancer were identified. The eligibility criteria for surgical resection of lung cancer with IPF were: a resting partial pressure of arterial oxygen > 60 mmHg; predicted postoperative forced expiratory volume in 1 s > 1.0 L; clinically stable and symptomless IPF; and complete resection possible. Of a total of 108 patients enrolled in this study, 17 (15.7%) with IPF developed second primary lung cancers. Thirteen of these patients underwent a second pulmonary resection, 2 patients received radiotherapy, 1 patient received chemotherapy, and 1 patient had best supportive care. The institutional review board approved this study (Niigata University, 2302) and waived the requirement for informed consent because the study was a retrospective review.

Radiologic assessment of the preoperative conventional chest computed tomography (CT) or high-resolution CT (HRCT) of all patients was performed to confirm the following criteria for IPF: 1) CT patterns compatible with IPF, as proposed by the American Thoracic Society and the European Respiratory Society [18], with bilateral reticular opacities and/or honeycombing predominant in the peripheral, subpleural, and basal locations; and 2) absence of known causes of pulmonary fibrosis, such as hypersensitivity pneumonitis, pneumoconiosis, sarcoidosis, eosinophilic pneumonia, lymphangioleiomyomatosis, drug-induced lung disease, and collagen vascular disease. One thoracic radiologist (HI) and one thoracic surgeon (SS) who were blinded to the clinical data evaluated the preoperative chest CT scans.

The medical records were reviewed to obtain the: demographic and clinical characteristics; chest CT scan findings; pulmonary function test results, including percent vital capacity (%VC) and percent forced expiratory volume in one second (FEV1%); surgical procedure; histologic findings; morbidity within 30 days of surgery; postoperative AE of IPF; and survival. AE was defined as: 1) onset within 30 days after pulmonary resection; 2) increasing respiratory distress; 3) newly developed fibrosis, ground glass opacity, or infiltrates on chest radiograph; 4) decrease in the resting partial pressure of arterial oxygen > 10 mmHg; and 5) the absence of heart failure or infectious lung disease [19]. A recently reported scoring system was used to assess the 30-day risk of AE onset after pulmonary resection in lung cancer patients with interstitial lung disease (ILD) based on seven risk factors, including a history of AE of ILD, preoperative steroid use, elevated serum sialylated carbohydrate antigen, KL-6 level, surgical procedure, usual interstitial pneumonia (UIP) pattern on CT scan, male sex, and low %VC [15].

Pathologic cancer stage was determined using the 7th edition of the International Union Against Cancer tumor-node-metastasis staging system [20]. Information was obtained for all survivors, either during office visits or by telephone interviews with the patient or a relative. The criteria for the diagnosis of multiple primary lung cancers were those described by Martini and Melamed [21] in 1975: 1) different histology or 2) same histology, if the disease-free interval between the two lesions was at least 2 years or development of a new neoplasm from an in situ carcinoma and occurrence of the second tumor in a different lobe or lung, provided that extrapulmonary metastases and lymphatic involvement common to both tumors were excluded. Tumors were designated as ‘synchronous’ when detected or resected simultaneously and as ‘metachronous’ when the second tumor was found some time later.

### Statistical analyses

The patients’ characteristics are expressed as counts and proportions; categorical variables were compared using the chi-squared test or Fisher’s exact test if there were 5 or fewer observations in a cohort. The Mann–Whitney *U*-test was used to compare quantitative parameters. Disease-free survival (DFS) was defined as the time from surgery to documented clinical progression or death. Overall survival (OS) was defined as the time from surgery to death. Prognosis was analyzed using the Kaplan–Meier method with the log-rank test. Differences were considered significant if the *P*-value was less than 0.05. All statistical analyses were performed using SPSS for Windows Version 22.0 (SPSS, Inc., Chicago, IL, USA).

## Results

### Patients’ characteristics

A total of 108 patients were diagnosed as having IPF based on conventional CT or HRCT findings, and, of them, 17 developed second primary lung cancers. Thirteen patients underwent repeated surgery, and 4 patients did not. The characteristics of the 13 patients are shown in Table 1, and those of the 4 patients are in Additional file 1: Table S1. The median interval between the initial surgery and the second surgery was 2 months (range, 1–3 months) in synchronous tumors, and 26 months (range, 8–68 months) in metachronous tumors. The study group included 11 men and 2 women. The mean age of the patients at the second surgery was 71.6 ± 8.6 years (range, 55–80 years). Six patients had synchronous tumors, and 7 patients had metachronous tumors. A total of 11 of 13 patients (84.6%) were heavy smokers (≥30 pack years). The mean %VC was 104.4% ± 16.1% before the initial surgery and 76.4% ± 19.7% before the second surgery. Regarding the AE risk score [15], 9 patients had intermediate risk and 4 patients had low risk at the initial surgery, but only 2 patients had intermediate risk at the second surgery. During both perioperative periods, IPF prophylaxis, such as steroids, sivelestat sodium hydrate, pirfenidone, and so on, was not given.

**Table 1:** Characteristics of patients

Variable^a^	Total = 13
Age, years	
at initial surgery	70.2 ± 8.4
at second surgery	71.6 ± 8.6
Sex	
male	11
female	2
Time interval in months, median (range)	
synchronous	2 (1–3)
metachronous	26 (8–68)
Type of multiple cancers	
synchronous	6
metachronous	7
Smoking history	
PY < 30	2
PY ≥30	11
FEV1%, mean	
at initial surgery	79.2 ± 6.8
at second surgery	84.5 ± 7.7
%VC, mean	
at initial surgery	104.4 ± 16.1
at second surgery	76.4 ± 19.7
KL-6 (U/ml), mean	
at initial surgery	693.8 ± 318.4
at second surgery	706.8 ± 377.2
AE risk score (initial/second)	
Low risk/Low risk	3
Low risk/Intermediate risk	1
Intermediate risk/Low risk	8
Intermediate risk/Intermediate risk	1

^a^Categorical data are expressed as numbers, and continuous data are expressed as means ± standard deviation

*PY* pack years, *FEV1* forced expiratory volume in 1 s, *VC* vital capacity, *KL-6* sialylated carbohydrate antigen KL-6, *AE* acute exacerbation

### Tumor location and type of surgical procedure

The location of the tumors was in the lower lobe in 9 patients at the initial surgery and in 8 patients at the second surgery. Of the 13 patients, 8 patients (61.5%) underwent lobectomy, 2 patients (15.4%) underwent segmentectomy, and 3 patients (23.1%) underwent wedge resection at the initial surgery. At the second surgery, 10 patients (76.9%) underwent wedge resection, and 3 patients (23.1%) underwent segmentectomy; none of the patients underwent lobectomy (Table 2).

**Table 2:** Location, surgical procedure, histology, and stage at initial and second surgeries

Variable	Initial No. (%)	Second No. (%)
Tumor location		
Upper	4 (30.8)	5 (38.5)
Lower	9 (69.2)	8 (61.5)
Surgical procedure		
Wedge	3 (23.1)	10 (76.9)
Segmentectomy	2 (15.4)	3 (23.1)
Lobectomy	8 (61.5)	0 (0)
Lymph node dissection		
None	3 (23.1)	12 (92.3)
Hilum	1 (7.7)	0 (0)
Mediastinum	9 (69.2)	1 (7.7)
Histology		
Ad	3 (23.1)	3 (23.1)
Sq	8 (61.5)	8 (61.5)
Ad-Sq	1 (7.7)	2 (15.4)
Sm	1 (7.7)	0 (0)
Pathological stage		
IA	3 (23.1)	4 (30.8)
IB	5 (38.5)	9 (69.2)
IIA	2 (15.4)	0 (0)
IIB	1 (7.7)	0 (0)
IIIA	2 (15.)	0 (0)

*ND* node dissection, *Ad* adenocarcinoma, *Sq* squamous cell carcinoma, *Ad-Sq* adenosquamous cell carcinoma, *Sm* small cell carcinoma

### Histologic diagnoses and tumor staging

As shown in Table 2, squamous cell carcinoma was the most common finding at the initial and second surgeries (*n* = 8, 61.5%, for both). Both initial and second tumors were squamous cell carcinoma in 4 patients and adenocarcinoma in 1 patient.

The pathologic stage of the initial tumor was stage I in 8 patients (61.5%), stage II in 3 patients (23.1%), and stage IIIA in 2 patients (15.4%). Ten patients underwent lymph node dissection.

At the second surgery, the clinical stage of the second tumor was stage IA in 12 patients and stage IB in only 1 patient. However, the pathologic stage of the second tumor was stage IA in 4 patients and stage IB in 9 patients. In all 8 cases, the reason for upstaging from IA to IB was pleural invasion. However, 12 patients (92.3%) underwent only sublobar resection without lymph node dissection.

### Postoperative acute exacerbation

**Fig. 1:**
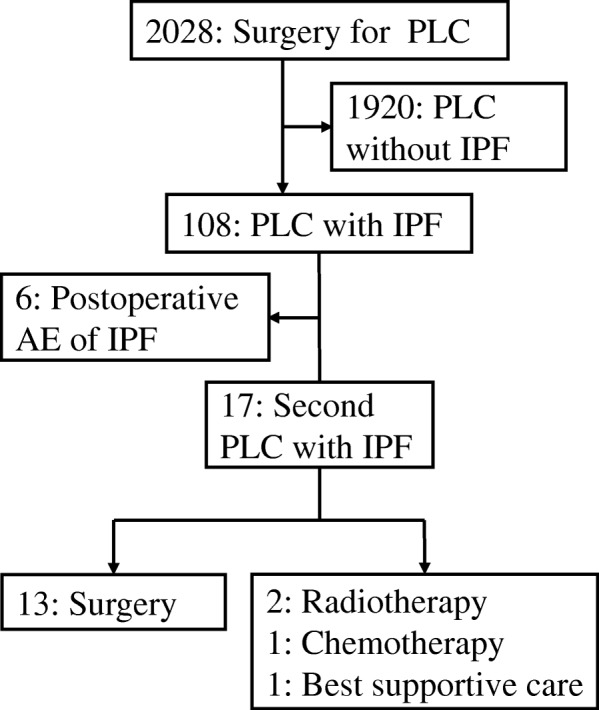
Flowchart of enrollment and investigations. Flowchart of enrollment and investigations after primary lung cancer (PLC) patients without idiopathic pulmonary fibrosis (IPF) were excluded; 108 patients were eligible for the analysis. Six patients had developed postoperative acute exacerbation (AE) at initial surgery. Seventeen patients with IPF developed second primary lung cancers. Thirteen of them underwent a second surgery

At initial surgery, 6 (5.6%) of 108 patients developed postoperative AE of IPF (Fig. 1). Table 3 shows the patients who developed postoperative AE of IPF at the initial or second surgery. Comparing the 6 patients at the initial surgery and the 3 patients at the second surgery, the AE risk score was significantly lower for the second surgery cohort than for the initial surgery cohort (*p* = 0.024).

**Table 3:** Clinical characteristics of patients with postoperative acute exacerbation after initial and second surgeries

Variable^a^	Initial *N* = 6	Second *N* = 3	*p* Value^b^
Age, years	71.3 ± 8.2	72.7 ± 8.4	1.000
Sex			
Male	6	3	NA
Female	0	0	
Smoking history (PY), mean	50.8 ± 8.3	78.7 ± 43.6	0.381
FEV1%, mean	81.9 ± 5.2	87.5 ± 11.2	0.393
%VC, mean	78.5 ± 9.8	64.4 ± 19.7	0.262
KL-6 (U/ml), mean	1204.4 ± 638.4	606.3 ± 279.2	0.143
AE risk score, mean	12.8 ± 1.2	9.0 ± 1.7	0.024
Low risk	0	2	
Intermediate risk	6	1	
Tumor location			
Upper	2	0	0.417
Lower	4	3	
Surgical procedure			
Wedge	0	1	0.333^c^
Segmentectomy	1	2	
Lobectomy	5	0	
Histology			
Ad	2	1	0.301
Sq	4	1	
Ad-Sq	0	1	
Pathological stage			
IA	0	1	0.392
IB	4	2	
IIA	1	0	
IIB	1	0	

^a^Categorical data are expressed as numbers, and continuous data are expressed as means ± standard deviation

^b^Values of *p* < 0.05 are significant

^c^Value is a comparison between wedge vs. segmentectomy and lobectomy

*NA* not available, *PY* pack year, *FEV1* forced expiratory volume in 1 s, *VC* vital capacity, *KL-6* sialylated carbohydrate antigen KL-6, *AE* acute exacerbation, *Ad* adenocarcinoma, *Sq* squamous cell carcinoma, *Ad-Sq* adenosquamous cell carcinoma

Comparison of the patients according to the presence or absence of postoperative AE of IPF at the second surgery is shown in Table 4. In this series, 3 patients developed and died of AE in the postoperative period (Table 5). The rate of %VC decrease was significantly higher in patients with AE than in patients without AE (*p* = 0.011). In all 3 patients with AE, tumor location was the lower lobe at the initial and second surgeries. On the other hand, development of AE was not significantly correlated with sex, types of multiple cancers, smoking history, KL-6, and combination of surgical procedures. Regarding the AE risk score that was proposed by Sato and colleagues at the second surgery [15], there was no significant difference between patients with and without AE. The risk of developing AE after the second surgery was low in 2 of 11 (18.2%) patients and intermediate in 1 of 2 (50%) patients. Over the same time period, the development of AE of IPF in 4 patients who did not undergo surgical treatment for second primary lung cancer was investigated, and it was found that no patients developed AE of IPF.

**Table 4:** Clinical characteristics of patients with and without acute exacerbation after the second surgery

Variable^a^	With AE (*n* = 3)	Without AE (*n* = 10)	*p* Value^b^
Sex			
Male	3	8	0.577
Female	0	2	
Type of multiple cancer			
Synchronous	1	5	0.563
Metachronous	2	5	
Smoking history			
PY < 30	0	2	0.577
PY ≥30	3	8	
FEV1%, mean	87.5 ± 11.2	83.3 ± 6.6	0.455
%VC, mean	64.4 ± 19.7	80.9 ± 19.0	0.234
Rate of %VC decrease from initial surgery, mean	35.9 ± 11.7	20.1 ± 5.6	0.011
KL-6 (U/ml), mean	596.3 ± 270.8	743.5 ± 413.8	0.583
AE risk score			
Low risk	2	9	0.423
Intermediate risk	1	1	
Combination of tumor location (initial/second)			
Lower/Lower	3	3	0.070
Other	0	7	
Combination of surgical procedure (initial/second)			
Wedge/Wedge	0	2	0.296
Lobectomy/Sublobar	3	5	
Other	0	3	

^a^Categorical data are expressed as numbers, and continuous data are expressed as means ± standard deviation

^b^Values of *p* < 0.05 are significant

*AE* acute exacerbation, *PY* pack years, *FEV1* forced expiratory volume in 1 s, *VC* vital capacity, *KL-6* sialylated carbohydrate antigen KL-6

**Table 5:** Clinical characteristics of patients with acute exacerbation after the second surgery

Variable	Case 1	Case 2	Case 3
Sex	Male	Male	Male
Age (years)	77	78	63
Time interval (months)	34	14	3
Type of multiple cancer	Metachronous	Metachronous	Synchronous
Smoking history (PY)	54	53	129
%VC at second surgery	87.1	52.6	53.4
Rate of %VC decrease	24.3	47.6	35.7
KL-6 (U/ml)	284	766	739
AE risk score	11	8	8
Tumor location (initial/second)	Lower/Lower	Lower/Lower	Lower/Lower
Surgical procedure (initial/second)	Lobectomy/Segmentectomy	Lobectomy/Wedge	Lobectomy/Wedge
Histology (initial/second)	Sq/Ad	Sq/Ad-Sq	Sq/Sq
Pathological stage	IA/IB	IB/IB	IA/IA

*PY* pack years, *VC* vital capacity, *KL-6* sialylated carbohydrate antigen KL-6, *AE* acute exacerbation, *Sq* squamous cell carcinoma, *Ad-Sq* adenosquamous cell carcinoma

### Survival

**Fig. 2:**
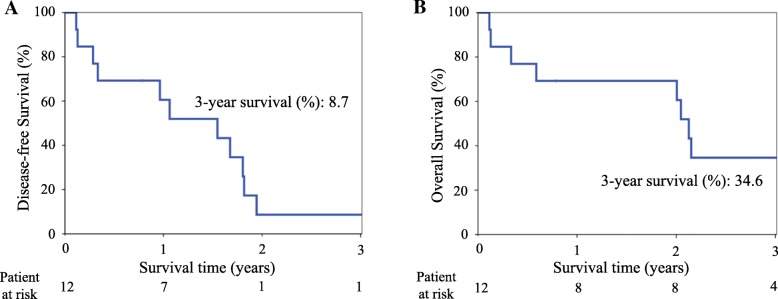
Outcomes in lung cancer patients with idiopathic pulmonary fibrosis after the second surgery. **a** The 3-year disease-free survival is 8.7%. **b** The 3-year overall survival is 34.6%

The median follow-up period after the second surgery was 24.9 months (range, 1.5–54.0 months). The disease-free survival (DFS) rates were 60.6% at 1 year and 8.7% at 3 years (Fig. 2a). Overall survival (OS) was 69.2% at 1 year and 34.6% at 3 years (Fig. 2b). Regarding the pattern of lung cancer recurrence in 8 patients, 4 patients developed intrathoracic disease (local), and 4 patients developed extrathoracic spread (distant). During the follow-up period, 11 patients (84.6%) died, and the most common cause was cancer-related; 7 patients died of recurrent lung cancer, and 1 patient died of additional metachronous lung cancer. The 3 other patients died of AE of IPF. Only 2 patients remained alive; one was free of relapse, while the other had local recurrence. Adjuvant therapy with oral tegafur-uracil after the second surgery was administered in 1 patient with stage IB adenocarcinoma.

## Discussion

The incidence of lung cancer is higher in patients with IPF than in those without IPF. Notably, Fujimoto and colleagues [22] reported a high incidence of second primary lung cancer in patients with IPF.

Lung cancer patients with IPF are more likely to develop severe morbidity and have poor outcomes after pulmonary resection. Considering the high recurrence rate and the poor prognosis for this patient population, operative indicators for major pulmonary resection in patients with lung cancer with IPF remain unclear. Kushibe and colleagues [23] reported that patients with IPF who had postoperative acute lung injury/acute respiratory distress syndrome had a significantly lower preoperative percent forced VC (%FVC) than those without such complications. IPF patients with a preoperative %FVC < 80% may not have an operative indication for lung cancer, and those with a preoperative %FVC ≥90% could have a good operative indication. Fujimoto and colleagues [22] suggested that patients with lung cancer invading the chest wall were excluded from surgery because chest wall resection is associated with major morbidity [24]. Pneumonectomy should be avoided for the same reason.

There have been several reports on the surgical outcomes of lung cancer patients with IPF [1, 7, 9, 15–17, 22, 23, 25]; however, to the best of our knowledge, the implications of repeated surgery in such patients have not been reported. The present study found a poor prognosis for patients with IPF even after complete repeated resection of lung cancer, with 3-year DFS and OS rates of 8.7% and 34.6%, respectively.

Recently, a large cohort study by Sato and colleagues [15] reported their derived scoring system for the 30-day risk of developing AE of IPF after pulmonary resection and classified patients into three risk groups (i.e., low, intermediate, and high). In the present study, higher rates of developing postoperative AE were found than cited in the previous report. According to Sato and colleagues, the predicted AE incidence was < 10% [95% confidence interval (CI): 0–10] in the low risk group and 10–25% (95% CI: 8.8–29.7) in the intermediate risk group, though 2 of 11 (18.2%) patients at low risk and 1 of 2 (50%) patients at intermediate risk developed and died of AE in the present study. The patients who developed postoperative AE at the initial surgery were compared with those who developed postoperative AE at the second surgery, and AE in the second surgery cohort developed with a significantly lower risk score than in the initial surgery cohort. The patients who did not undergo surgical treatment for second primary lung cancers were also examined, and no patients developed AE of IPF. Although the present study sample was very small, repeated surgery for lung cancer in patients with IPF could be a risk factor for AE.

The etiologic agents of AE of IPF after pulmonary resection remain unclear. Sakamoto and colleagues [26] reported the possible factors contributing to AE after surgery in patients with IPF: 1) high activity of the disease prior to the surgery; 2) oxygen supplementation at a high concentration during surgery; 3) surgical stress including mechanical ventilation-related lung injury; 4) complicating respiratory infections; 5) postoperative reduction of steroid dose; and 6) medications (anesthesia, anticancer drugs, and so on). Misthos and colleagues [27] investigated the possibility of an association between oxygen radical toxicity and the occurrence of AE of IPF. They found the following: 1) lung re-expansion after one-lung ventilation (OLV) provoked severe oxidative stress; 2) the degree of oxygen-derived free radicals generated was associated with the duration of OLV; 3) patients with lung cancer had higher production of oxygen-derived free radicals than the normal population; 4) tumor resection removes a large oxidative burden from the organism; 5) mechanical ventilation and surgical trauma are weak free radical generators; and 6) manipulated lung tissue is also a source of oxygen-derived free radicals, not only intraoperatively, but also for several hours later.

The present analysis showed that the rate of %VC decrease was significantly higher in patients with AE than in those without AE. %VC has been considered a reliable marker of fibrotic change [11, 13], and some previous studies [23, 25] reported that %VC had a significant and independent association with the development of AE. In the present study, the %VC of 1 patient with AE was not lower than 80%, but the rate of %VC decrease was 24.3%. Therefore, the AE incidence at the second surgery for lung cancer in patients with IPF is associated not only with a low %VC, but also with a high rate of %VC decrease. Also, among the pulmonary function tests, %DLCO has been considered a survival predictor [7, 28] and a reliable indicator of fibrotic change [12, 13, 29]. However, it was not included in the present study because the values of only 3 of 13 patients were available.

In the present study, the reason for the poor prognosis was a high rate of cancer recurrence, aside from development of AE. Although all patients were clinical stage IA or IB at the second surgery, 8 of 10 patients had cancer recurrence, except those who died of AE. Saito and colleagues [16] reported that the 5-year survival of lung cancer stage IA patients with IPF was 54.2%. Watanabe and colleagues [9] reported a 5-year survival of 61.6% after pulmonary resection for patients with stage I. Sato and colleagues [17] reported that the 5-year survival rates were 59% and 42% for pathologic stages IA and IB, respectively. Watanabe and colleagues [9] and Okamoto and colleagues [7] suggested that the frequency of cancer recurrence was higher in patients with IPF than in those without. Sato and colleagues [17] reported that recurrence was the main cause of death and posed a risk that was about twice as high as that of respiratory disorders; they underscored the importance of oncologic control for survival. In the present study, sublobar resection was performed in all patients, and lymph node dissection was performed in only 1 patient at the second surgery. Sato and colleagues [17] noted that stage IA patients who underwent wedge resection had a prolonged survival and were less likely to develop AE of IPF, but they had a higher cancer recurrence rate than patients who underwent lobectomy. Furthermore, patients who underwent segmentectomy had less favorable oncologic outcomes than patients who underwent lobectomy. At the second surgery, accurate pathologic staging might not be possible because lymph node dissection was not performed in almost all patients. Needless to say, it was necessary to consider the influence of lung cancer recurrence not only in the second surgery, but also at the initial surgery. Actually, of the 5 of 8 patients who developed recurrence, 3 were stage II and 2 were stage III at the time of the initial surgery.

With regard to distinguishing multiple primary lung cancers from primary lung cancer with intrapulmonary metastasis, the possible effects of multiple lung tumors, as defined by Martini and Melamed [21], were considered. Although it is important to distinguish a second primary cancer from local recurrence or metastatic disease, this is sometimes difficult and even impossible. Girard and colleagues [30] considered that biologic examinations could be performed, assuming that the independent tumor clones harbor distinct mutations. In the present study, the same histologic diagnoses at the initial and second surgeries were seen in 4 patients with squamous cell carcinoma and in 1 patient with adenocarcinoma. Among them, 2 patients with squamous cell carcinoma and the patient with adenocarcinoma were investigated, but they were negative for epidermal growth factor receptor mutation at both the initial and second surgeries.

Squamous cell carcinoma had a higher prevalence than the other histopathological types among the patients with IPF in the present study, consistent with the previous reports. The cause of the high prevalence of squamous cell carcinoma in IPF patients remains unclear. Song and colleagues [31] found many foci of squamous metaplasia in honeycombing epithelium, and Hironaka and Fukuyama [32] reported that IPF patients with lung cancer showed more frequent foci of squamous metaplasia than IPF patients without lung cancer. Calabrese and colleagues [33] reported the overexpression of squamous cell antigen, a serine protease inhibitor typically expressed by dysplastic and neoplastic cells of epithelial origin, more often in squamous cell tumors, in IPF. These pathological findings support the notion that IPF may be a precursor to the development of squamous cell carcinoma.

### Limitations

The present study had some limitations. First, only 3 patients developed AE of IPF at the second surgery; this number could be insufficient to predict the tendency for developing AE. Second, the present study included only IPF patients who underwent surgical procedures for multiple primary lung cancers and did not include IPF patients with multiple primary lung cancers who did not undergo surgical intervention. Therefore, the implications of a second surgery for lung cancer patients with IPF remain unclear. Third, this was a retrospective, two-institution study with a limited sample size. Further studies should be conducted to identify who among the patients with IPF who had undergone a first pulmonary resection for lung cancer could benefit from interventions, including surgery, chemotherapy, and radiotherapy, for the multiple primary lung cancers.

## Conclusions

Postoperative development of AE and the long-term survival of patients with a second primary lung cancer with IPF who underwent repeated surgery were investigated. The main cause of their poor prognosis was cancer death, possibly related to sublobar resection. Repeated surgery for patients with lung cancer and concomitant IPF could increase the risk of AE development despite limited surgery. The rate of %VC decrease might be correlated with the incidence of AE. Although these results demonstrated that surgical intervention for multiple primary lung cancers might be contraindicated in patients with IPF, selection of patients who may benefit from such treatment is very important. To confirm these findings, a large, long-term, multi-center surveillance study will be required.

## Additional file

Additional file 1: **Table S1.** Clinical characteristics of patients without surgical treatment for second primary lung cancer. (DOCX 13 kb)

## Abbreviations

%VC: Percent vital capacity
AE: Acute exacerbation
CI: Confidence interval
CT: Computed tomography
FEV1%: Percent forced expiratory volume in one second
HRCT: High-resolution computed tomography
ILD: Interstitial lung disease
IPF: Idiopathic pulmonary fibrosis
UIP: Usual interstitial pneumonia
