# Supplementary material for: Survival after repeated surgery for lung cancer with idiopathic pulmonary fibrosis: a retrospective study
Source: BMC Pulm Med. 2018 Aug 10;18:134. doi: 10.1186/s12890-018-0703-8 (PMC6086038; doi:10.1186/s12890-018-0703-8)
Supplement: Supplementary file 1 — Table S1. Clinical characteristics of patients without surgical treatment for second primary lung cancer. (DOCX 13 kb) [file 12890_2018_703_MOESM1_ESM.docx]

| Table S1. Clinical characteristics of patients without surgical treatment for second primary lung cancer | | | | |
| --- | --- | --- | --- | --- |
| Variable | Case 1 | Case 2 | Case 3 | Case 4 |
| Sex | Male | Male | Male | Male |
| Age (years)^a^ | 72 | 70 | 77 | 76 |
| Time interval (months) | 30 | 63 | 10 | 14 |
| Smoking history (PY) | 135 | 45 | 72 | 70 |
| Tumor location (initial/second) | Upper/Lower | Lower/Upper | Upper/Lower | Upper/Lower |
| Tumor site | Contralateral | Contralateral | Ipsilateral | Ipsilateral |
| Surgical procedure at initial | Lobectomy | Wedge | Lobectomy | Wedge |
| Histology (initial/second) | Ad/Sq | Sq/Sm | Sq/Ad | Sq/NOS |
| Stage (initial/second) | IIIA/IA | IB/IIA | IIIA/IB | IB/ |
| Treatment at second PLC | Radiotherapy | Chemotherapy | Radiotherapy | BSC |
| Survival^b^ (months) | Alive (38) | Cancer death (14) | Cancer death (4) | Cancer death (14) |
| Reason for another treatment | Poor PS | Advanced Stage | Poor PS | Refusal |
| ^a^Age at the time of second primary lung cancer treatment  ^b^Period from the second primary lung cancer treatment  PY = pack years; Ad = adenocarcinoma; Sq squamous cell carcinoma; Sm = small cell carcinoma; NOS = not otherwise specified; PLC = primary lung cancer; BSC = best supportive care; PS = performance status | | | | |
